# Structural Stability Assessment for Optimal Order Picking in Box-Stacked Storage Logistics

**DOI:** 10.3390/s25041085

**Published:** 2025-02-11

**Authors:** Haegyeom Choi, Hojin Yoon, Eunbin Jung, Donghun Lee

**Affiliations:** Department of Mechanical Engineering, Soongsil University, Seoul 06978, Republic of Korea; choihg@soongsil.ac.kr (H.C.); hojin98@soongsil.ac.kr (H.Y.); enbang@soongsil.ac.kr (E.J.)

**Keywords:** structural stability assessment, box-stacked storage, optimal order picking, CNN

## Abstract

This study proposes a method for time-efficient order picking based on a structural stability assessment (SSA) when target boxes inside box-stacking storage (BSS) on multi-layer racks are removed. This method performs optimal order picking by generating a path to directly pick the target box without first picking the upper boxes in the BBS, if it is possible to pick the target box directly. The SSA algorithm generates images of the complement structure by removing the target box within BBS and uses them as input data for the CNN model to evaluate the stability of the structure. To create the CNN model, we generated a dataset using CoppeliaSim simulation, considering the size and shape of the overall structure of the BBS, the size and number of each box, and the number of target boxes. The accuracy of the generated CNN model was 95.1% on test data, while it achieved 97% accuracy when using real-world data. This validation process confirmed that the algorithm can be effectively applied to real BBS logistics environments to perform optimal order picking.

## 1. Introduction

Global companies in traditional industries such as manufacturing and logistics have been increasingly adopting robotics and automation technologies to address productivity challenges and enhance competitiveness [1]. In particular, the logistics market has experienced explosive growth in recent years due to the pandemic, and the changing demands of consumers have driven the need for highly advanced automation systems. Consequently, logistics development is evolving toward “Logistics 4.0”, which is defined as a logistics system that supports industrial and trade development through digital technology while ensuring sustainable satisfaction of individualized customer demands without incurring additional costs, in alignment with the principles of Industry 4.0 [2]. Warehouse Management Systems (WMSs) are utilized to enable the efficient operation of logistics warehouses, and order picking—one of the primary processes of WMSs—is an essential step in fulfilling customer orders. This process accounts for the largest share of labor costs within logistics warehouses, comprising approximately 55% of total labor expenses [3]. To alleviate the economic burden and enhance efficiency, numerous studies have been actively conducted to improve the order picking process, which holds the highest proportion of labor costs [4].

**Figure 1:**
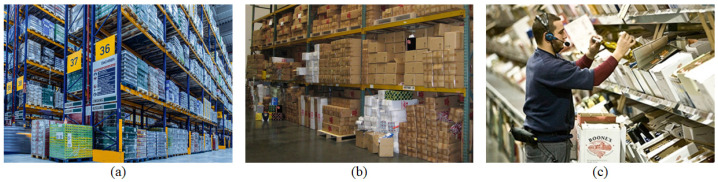
The results of classifying logistics warehouses by their operational purpose are as follows: (**a**) storage warehouses, (**b**) distribution centers, (**c**) fulfillment warehouses.

As shown in Figure 1, the environments where order picking is applied within logistics warehouses can be classified into three categories based on operational objectives: storage warehouses [5], distribution centers [6], and fulfillment warehouses [7]. Storage warehouses focus on efficiently storing large quantities of goods that require long-term storage, primarily managing items on a pallet basis to optimize space utilization and prevent inventory damage. Distribution centers emphasize the rapid inbound and outbound handling of most goods on a box basis to facilitate efficient distribution to various destinations. These centers primarily operate in a Business-to-Business (B2B) context, requiring high inventory turnover and fast processing speeds. On the other hand, fulfillment warehouses are designed for Business-to-Customer (B2C) operations, focusing on processing individual customer orders, and emphasizing rapid packing and delivery. This study aims to improve the order picking process within distribution centers, which is a BBS that manages products by box, enabling fast processing and efficient distribution to various destinations.

Methods to improve the order picking process can be categorized into three areas: warehouse layout and structure [8,9], picking process [10,11], and storage allocation [12,13]. Improvements in warehouse layout and structure aim to optimize the warehouse’s layout by utilizing various simulation scenarios to minimize workers’ travel paths, reduce travel time, and enhance space utilization. Improvements in the picking process involve automation and assistance technologies, such as pick-to-light and voice picking systems, to enhance picking accuracy and speed, as well as optimizing picking path planning. Enhancements in storage allocation focus on optimizing inventory placement within the warehouse, positioning popular items in optimal locations to reduce workers’ travel time. Given that distribution centers require high inventory turnover and fast processing speeds, improving picking efficiency is essential. Therefore, this study focuses on optimizing the picking process to enhance the order picking efficiency within distribution centers.

In a distribution center environment where most goods are managed in box units, Jacopo Aleotti’s research [14] developed an end-effector tool to optimize the picking process. This tool enables the recognition of box positions on the top layer and precisely identifies the gaps between boxes, allowing boxes to be picked using a sliding mechanism without lifting them. As a result, it is possible to pick target boxes without disassembling the entire stacked structure. However, when a target box is specified, the upper boxes above the target must necessarily be picked beforehand. In Riccardo Caccavale’s research [15], efficient paths are generated to pick a target box without removing all the upper boxes. However, this approach requires prior knowledge of the configuration information, including the position and size of all boxes. Even in cases where the structure allows for picking the target box without removing the upper box directly above it, the upper box is still picked first before accessing the target box. To generate an optimal picking path for a target box while avoiding the removal of upper boxes, it is crucial to assess the stability of the complementary structure that remains after the target box is removed.

To assess the stability of a complement structure, two approaches can be considered: using physical feasibility based on complete configuration information of all boxes, and leveraging physical intuition, which mimics human instincts. In Teemu Linkosaari’s research [16], physical feasibility was employed to evaluate structural stability by utilizing pre-known configurations of all boxes. This approach included methods such as calculating the maximum overhang, edge contact support, and the center of gravity for each layer of boxes. However, in logistics warehouses, uncertainties often make it challenging to obtain precise information about the position and dimensions of all boxes, necessitating the use of physical intuition methods. Haozhi Qi’s research [17] introduced the Region Proposal Interaction Network (RPIN), which predicts the trajectories of individual objects in a stacked structure to determine structural stability. While this method effectively predicts stability in structures where objects are stacked in simple vertical arrangements, it cannot be applied to more complex logistics environments, such as distribution centers, where boxes are stacked in multi-directional arrangements (left, right, top, bottom). Thus, alternative methods are required to address the unique challenges of such environments.

Therefore, this study aims to generate an optimal picking path for a target box by creating a SSA to evaluate the stability of the complement structure after the removal of the target box in a distribution center environment, where goods are stacked in multi-directional arrangements. First, data are created, and the model is trained to create a CNN model for the SSA algorithm. Subsequently, a real-time test environment resembling a distribution center, a BBS environment, will be set up. In this environment, the YOLOv8-seg model will detect boxes exclusively. To select the target box from the detected boxes, a previously developed direct teaching interface will be employed to remotely designate the target box. Once the target box is selected, the stability of the complement structure, following its removal, will be assessed, and an optimal picking path for the target box will be generated based on this evaluation.

The remainder of this paper is organized as follows: Section 2 presents the detailed methodology for generating the optimal path using the proposed SSA. Section 3 discusses the results of the proposed method, and Section 4 concludes the paper with a summary of findings and implications.

## 2. Methods

### 2.1. Overall Framework

**Figure 2:**
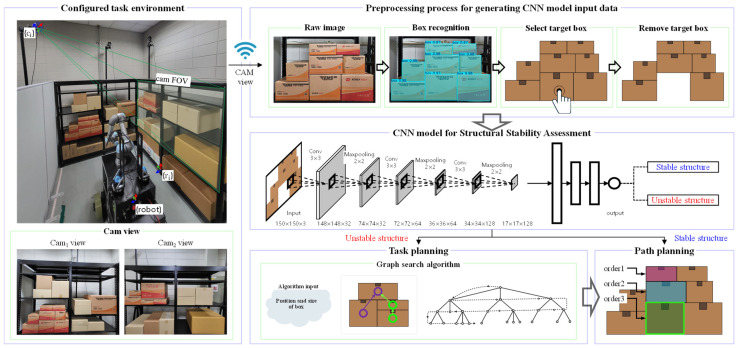
An overall framework for generating optimal paths for target box picking based on the SSA method.

The overall framework for generating the optimal path for picking a target box using the proposed method is shown in Figure 2. Racks were installed to construct an environment similar to a distribution center, which is a BBS environment, and various boxes were stacked on each rack to create the test environment. However, in a real environment, there may not be only boxes with regular shapes and similar materials, as shown in Figure 2, and the weight distribution may be different. However, most boxes handled in a distribution center generally have the shape of a rectangular solid with similar materials, and considering the space utilization of each box, the contents in each box will be full, so the center of gravity will be in the center. Therefore, the BBS environment was constructed without considering the special situation above. A YOLOv8-seg-based segmentation model was developed in this environment to recognize only the boxes. Using a pre-developed direct teaching interface [18], the target box is selected, and a complement structure is generated by virtually removing the target box. This complement structure is used as input data for the CNN model to perform the SSA. To train the CNN model, numerous images of complement structures with stability labels are required. Since it is impractical to create all possible complement structures in a real-world setting, the dataset is generated using CoppeliaSim 4.6.0 simulations. Random complement structures are created in the simulation environment, and a physics engine is used to label each structure as stable or unstable. This labeled dataset is then used to train the CNN model, which evaluates the stability of complement structures after the removal of the target box. If the complement structure is deemed stable, it indicates that the target box can be picked directly, and an optimal picking path is generated accordingly. If the structure is unstable, a pre-developed graph G-based algorithm is utilized for task planning to generate the shortest path for picking the target box while maintaining structural stability.

### 2.2. Box Recognition Based on YOLOv8-seg

To apply the proposed algorithm within a distribution center, it is essential to accurately recognize only the boxes from images captured by a camera positioned to face the racks. To achieve this, the YOLO model, known for its high object segmentation performance [19], was selected, and since the boxes had to be segmented in real time, the YOLOv8 model [20], which has good real-time performance among the YOLO models, was ultimately utilized. To train the YOLOv8-seg model, approximately 1300 images were generated by varying the distance between the camera and the rack, adjusting the camera’s orientation to adjust the incoming contrast of the camera and the angle at which it faces the boxes, and changing the arrangement of the boxes. In each generated image, the boxes’ sides, top, and front faces were visible. However, to evaluate the stability of complement structures, images must be created based on the front faces of the boxes. To address this, the smart polygon feature of Roboflow was used to label only the front faces of the boxes in all 1300 images, resulting in the final dataset. Then, data augmentation was performed by randomly adjusting the contrast ratio and adding random noise to ensure the robustness of the model, expanding the dataset to a total of 2100 images. Of these, 80% were used for training and the remaining 20% for validation to train the YOLOv8-seg model effectively.

### 2.3. Structural Stability Assessment

#### 2.3.1. Dataset for Training the CNN Model for SSA

To create the CNN model for evaluating the stability of complement structures after removing a target box using the direct teaching interface, a dataset containing images of various stacked box structures and stability labels for each structure is required. This dataset was generated using CoppeliaSim simulation by stacking boxes of various sizes and randomly selecting target boxes to create a variety of complement structures. However, since there are differences between simulation and reality such as friction, gravity, and collision, the MuJoCo physics engine [21] with the highest simulation accuracy was utilized to reduce these differences. In addition, since the movement of the robot is not learned through simulation and applied to reality, but rather a virtual camera is simply created in the simulation to create image data for CNN model training, there will be almost no difference from reality. To generate various structures in this environment, CoppeliaSim, five control factors were utilized: the overall structure and size of the BBS, the size and number of each box, and the number of target boxes. In addition, noise factors such as the weight and center of gravity of the box may exist, but the weight is proportional to the size of the box, and the center of gravity is assumed to be the center, as mentioned before. As shown in Table 1, the generated structures are divided into four cases, and various random configurations were generated.

**Figure 3:**
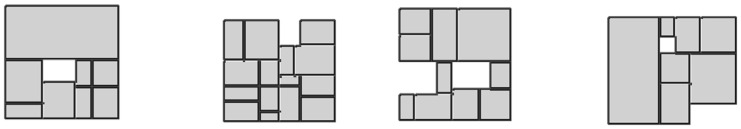
Sample image from the dataset for creating a CNN model using Coppelia simulation.

The final generated BBS structure is shown in Figure 3. We created a structure like this, took images through a virtual camera, and then identified only the front of the box to generate the final image for the CNN model input. Next, the physics engine was activated to simulate the behavior of the boxes. If the boxes moved and caused the structure to collapse, the structure was labeled as unstable. Conversely, if the structure remained intact without collapsing, it was labeled as stable. This process resulted in a dataset of approximately 40,000 images, evenly distributed between the two labels, with around 20,000 stable and 20,000 unstable structures.

#### 2.3.2. CNN Model Learning

**Figure 4:**
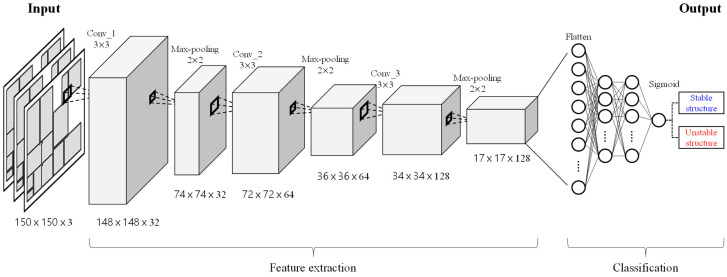
CNN model architecture for evaluating complement structural stability.

To train the CNN model using the dataset generated from the CoppeliaSim simulation, a CNN model with the architecture shown in Figure 4 was designed. The model is structured to sequentially perform feature extraction and classification, processing RGB images as input. The model’s architecture consists of the following key layers:

1. Convolutional Layers: These layers use filters of size 3 × 3, with the number of filters increasing progressively across layers (32, 64, and 128). These layers extract spatial features from the input images.
2. Max Pooling Layers: Placed after each convolutional layer, these layers reduce the spatial dimensions by half, focusing on the most prominent features while minimizing computational complexity.
3. Flatten Layer: The feature maps produced by the convolutional and pooling layers are converted into a 1D vector, preparing the data for classification.
4. Fully Connected Layers: The 1D vector output from the flatten layer is fed into the fully connected layers, which perform the final classification of the input image as either stable or unstable.

This architecture is optimized for processing the complement structure dataset, ensuring efficient feature extraction and robust classification.

In the fully connected layer, 512 nodes are used with the ReLU activation function to learn high-dimensional representations. To prevent overfitting, a dropout technique is applied with a dropout rate of 50%. The output layer employs the sigmoid activation function to return probabilities for binary classification, distinguishing between stable and unstable structures. Additionally, data augmentation techniques were applied to improve the generalization performance of the model on the training data. These augmentations enhance the model’s robustness by simulating variations and scenarios encountered in real-world environments. With this design, incorporating data augmentation and dropout, the model demonstrates strong generalization performance, making it suitable for application in distribution centers where diverse stacking scenarios are encountered.

### 2.4. Task Planning and Path Planning

**Figure 5:**
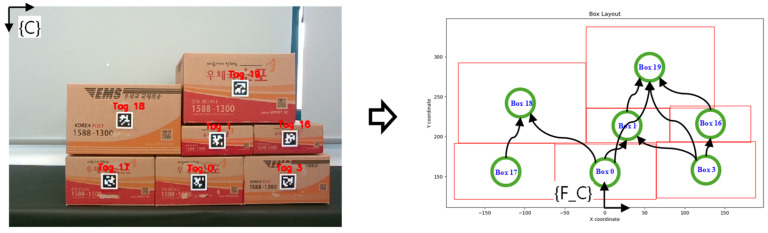
Graph G-based algorithm for optimal path generation.

After the stability assessment of the complement structure is conducted using the CNN model, the next step is to generate the optimal path for picking the target box based on the assessment results. If the complement structure is deemed unstable, directly picking the target box is infeasible due to the risk of structural collapse. In such cases, additional task planning is required. As shown in Figure 5, task planning involves defining each box as a node in a graph and representing the positional relationships between boxes as edges connecting these nodes. Utilizing a previously developed graph G-based algorithm [18], the shortest path is generated to pick the target box while maintaining the stability of the overall structure. On the other hand, if the complement structure is determined to be stable, no additional task planning is necessary. In this scenario, a direct picking path for the target box is generated, maximizing the efficiency of the order picking process. The overall pseudo code for this approach is shown in Algorithm 1, and it can be applied practically in complex logistics environments by ensuring both safety and operational efficiency of the order picking process by utilizing this approach.
**Algorithm 1.** Pseudo code for optimal path generation using CNN model and graph G algorithm**Algorithm** Task planning**Input**: CNN_model, complement_structure_image, target_box, G_AL ^1^1stability_assessment_result = CNN_model(complement_structure_image)2If stability_assessment_result == True:3  path = target _box4else:5  path = G_AL(target_box)**Output**: path^1^ AL: Algorithm

## 3. Experiment and Results

### 3.1. Experimental Environment Setup and Box Recognition

**Figure 6:**
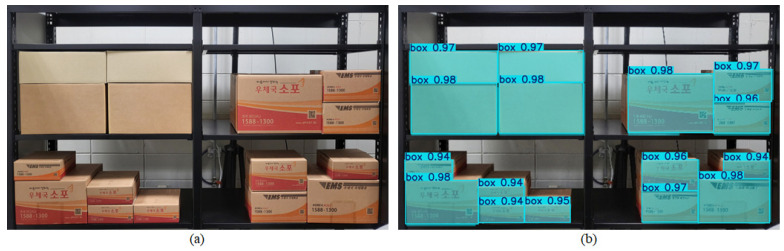
Recognition of the front of the box, which is an object in the distribution center, which is a BBS environment: (**a**) stacking of boxes in a distribution center, (**b**) recognized front of the boxes.

To construct an environment similar to a distribution center, which is a BBS environment, racks were installed, and boxes were stacked on the racks, as shown in Figure 6a. This setup was used to conduct experiments on the method for generating optimal picking paths using the CNN model. To recognize the boxes on the racks, a YOLOv8-seg-based segmentation model, trained specifically for this purpose, was utilized. The results of this segmentation are shown in Figure 6b. These results demonstrate the model’s ability to accurately recognize only the front faces of the objects (boxes) in a complex distribution center environment. Using the bounding box coordinates of the recognized front faces, input data for the physical intuition-based CNN model will be generated.

### 3.2. Generate CNN Model Input Data

**Figure 7:**
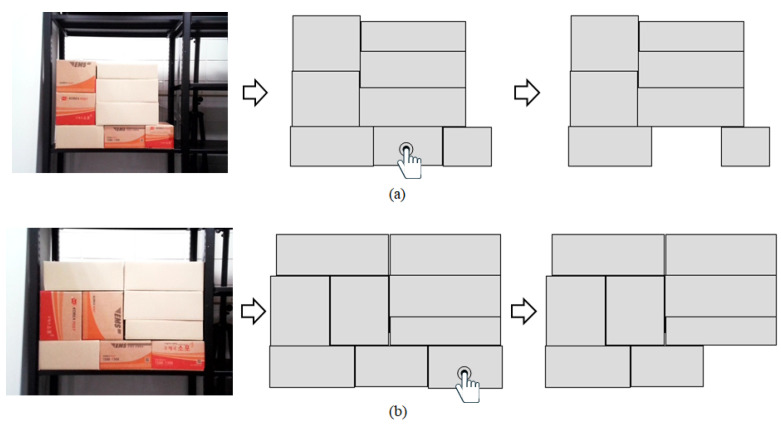
Generate CNN model input image using bounding box coordinates of recognized boxes: (**a**) When the lower central box is selected as the target box, (**b**) When the lower right box is selected as the target box.

To create input data for the CNN model using the bounding box coordinates of each recognized box, the process begins by extracting only the regions corresponding to the boxes from the input image, based on the bounding box coordinates. This results in a new image that focuses solely on the boxes, as shown in the center of Figure 7. Using the direct teaching interface, the target box is then selected, and once the target box is removed, the resulting image, excluding the target box, is shown on the right side of Figure 7. This final image is utilized as input data for the CNN model. Through this process, the YOLOv8-seg-based segmentation model is employed to recognize boxes within the distribution center accurately, and the bounding box coordinates are used to extract images of the regions containing the boxes. By selecting and removing the target box using the direct teaching interface, the final image is prepared as input for the CNN model, ensuring that the method seamlessly integrates recognition, selection, and data preparation steps for stability assessment.

### 3.3. CNN Model for SSA

**Figure 8:**
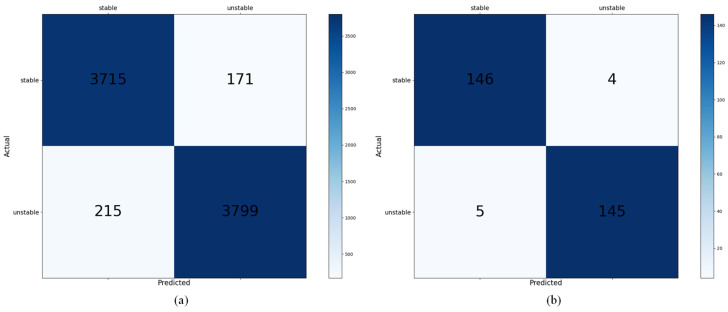
Confusion matrix of a CNN model: (**a**) results using test data, (**b**) results using real-world data.

The CNN model was trained to assess the stability of complement structures using input images where the target box was removed, as recognized by the YOLOv8-seg-based segmentation model. The training data consisted of approximately 40,000 samples that were labeled through CoppeliaSim simulation and generated in advance, 80% of which were used as training data, and the remaining 20% were used as validation data. The training result using the training data was 95.4%, and the validation result using the validation dataset showed an accuracy of 94.4%, proving that the model can perform an excellent stability assessment. Additionally, an approximately 10,000-test dataset was generated using CoppeliaSim to evaluate the model. As shown in Table 2, the accuracy achieved using the test dataset was 95.1%, and the calculated confusion matrix is presented in Figure 8a.

**Figure 9:**
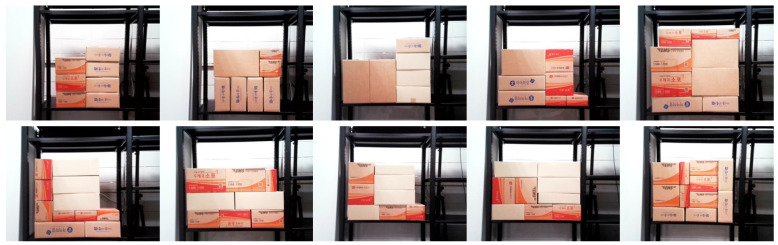
The results of configuring the BBS in 10 different scenarios to evaluate the SSA in a real-world scenario.

To test the model using images generated in a real-world scenario, the preprocessing steps described in Section 3.1 were applied to create input data for the CNN model. In the real environment, as shown in Figure 9, a total of 10 structures were built for testing purposes. In each structure, 1–2 target boxes were selected, resulting in the generation of 300 complement structure images. Using the generated images to test the CNN model, the results showed an accuracy of 97%, as seen in Table 2, and the calculated confusion matrix can be found in Figure 8b. These results validate the capability of the model to perform real-time stability assessments with high accuracy in complex logistics environments such as distribution centers.

### 3.4. Generate Path for Optimal Order Picking

**Figure 10:**
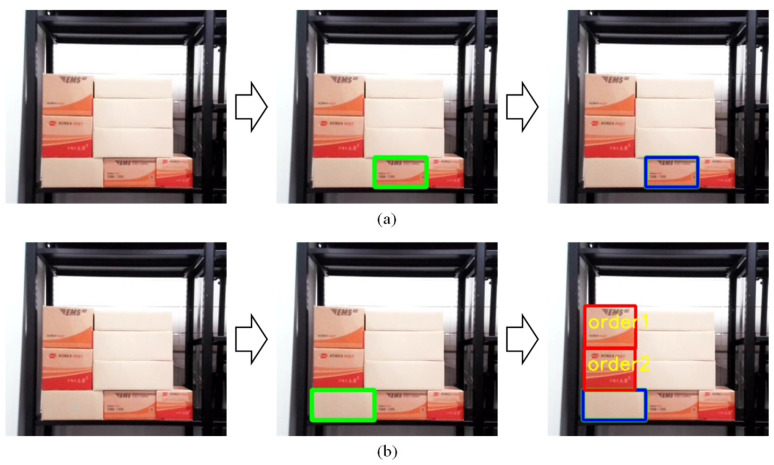
Optimal path generation based on the CNN model: (**a**) optimal path generation result when the complement structure is stable, (**b**) optimal path generation result when the complement structure is unstable.

The target box was selected using the direct teaching interface, and the complement structure was generated to evaluate its stability using the CNN model. Based on the evaluation results, the optimal picking path was determined. If the complement structure, excluding the target box, was deemed stable, as shown in Figure 10a, the target box could be picked directly. In this case, a direct picking path for the target box was generated to maximize efficiency. Conversely, if the complement structure was assessed as unstable, as illustrated in Figure 10b, direct picking of the target box was not possible due to the risk of structural collapse. In this scenario, a path was generated to first pick the upper boxes above the target box. A pre-developed graph G-based task planning algorithm was used to achieve this. As shown in the far-right illustration of Figure 10b, this algorithm generated a safe and efficient path, ensuring the stability of the structure while allowing the target box to be picked successfully.

To evaluate the efficiency of the CNN-based path generated by the proposed structural stability assessment model and the path simply generated based on graph G, we built BSS structures in CoppeliaSim and randomly selected target boxes. First, approximately 300 BSS structures were created in CoppeliaSim, and one or two target boxes were randomly selected from each structure. Paths for picking the target boxes were then generated using each method. When generating the picking path using the CNN-based structural stability assessment model, a total of 552 upper boxes needed to be picked before the target boxes. In contrast, the method using graph G required picking 844 upper boxes before the target boxes. Using this result and the picking speed calculation method proposed in AutoStore(Nedre Vats, Norway) [22], it can be seen that using the CNN model results in a picking speed 1.6 times faster than when using graph G.

## 4. Conclusions

This study presented a comprehensive framework to optimize the order picking process in a distribution center environment by employing a CNN model for SSA. The framework integrates YOLOv8-seg-based segmentation for box recognition, a direct teaching interface for intuitive target box selection, and a graph G-based task planning algorithm for safe and efficient picking path generation. The proposed approach addresses the critical challenges of structural stability and operational efficiency in logistics environments characterized by complex stacking configurations.

The CNN model, trained using a dataset generated through simulations in CoppeliaSim, demonstrated a high-test accuracy of 95.1% in stability assessments. The dataset, consisting of approximately 40,000 labeled images, provided diverse scenarios for model training, enabling robust generalization to real-world conditions. Real-time experiments conducted in a simulated distribution center environment further validated the model’s performance. By accurately recognizing box front faces and assessing complement structure stability, the system differentiated between stable and unstable scenarios. In cases where the complement structure was determined to be stable after the removal of the target box, the system generated a direct picking path, allowing for efficient order fulfillment. Conversely, when the complement structure was unstable, the graph G-based task planning algorithm was employed to prioritize the removal of upper boxes before safely picking the target box. This dual approach not only ensures the structural stability of stacked boxes, but also optimizes efficiency by minimizing unnecessary work, providing a process that is 1.6 times faster than the traditional order picking process. The results of this study highlight the practical applicability of the proposed framework in dynamic logistics environments, such as distribution centers, where high inventory turnover and fast processing speeds are essential. By combining high-performance object recognition, stability assessment, and task planning, the framework effectively addresses the demands of modern logistics operations. Future work could explore further optimization of task planning algorithms and the integration of more advanced simulation environments to improve the adaptability and scalability of the proposed method in broader logistics scenarios. We also plan to conduct additional research on algorithms that can be applied in situations other than those included in this study.

## Figures and Tables

**Table 1:** Five control factors for generating various complement structures.

	Overall Frame of BBS	Size of BBS	Size of Each Box	Total Number of Each Box	Number of Target Boxes
Case 1	Rectangular	W: 20~30 H: 20~22	W: 1.5~7.2 H: 1.5~5.3	1~16	1~3
Case 2	Rectangular	W: 20~30 H: 20~22	W: 1.5~10.3 H: 1.5~7.5	1~8	1~2
Case 3	L-shaped	W: 20~30 H: 20~22	W: 1.5~7.2 H: 1.5~5.3	1~16	1~3
Case 4	L-shaped	W: 20~30 H: 20~22	W: 1.5~10.3 H: 1.5~7.5	1~8	1~2

**Table 2:** The results of the CNN model using test data and real-world data as input.

Test and Real-World Data Assessment [%]		
Stable Structure	Unstable Structure	Total
95.6/97.3	94.6/96.7	95.1/97

## Data Availability

The original contributions presented in the study are included in the article; further inquiries can be directed to the corresponding authors.
